# Filling the gap despite full attention: the role of fast backward inferences for event completion

**DOI:** 10.1186/s41235-018-0151-2

**Published:** 2019-01-28

**Authors:** Frank Papenmeier, Alisa Brockhoff, Markus Huff

**Affiliations:** 1 Department of Psychology, University of Tübingen, Schleichstr. 4, 72076 Tübingen, Germany; 2 Department of Research Infrastructures, German Institute for Adult Education, Heinemannstraße 12-14, 53175 Bonn, Germany

**Keywords:** Event cognition, Backward inferences, Predictive perception, Event segmentation

## Abstract

The comprehension of dynamic naturalistic events poses at least two challenges to the cognitive system: filtering relevant information with attention and dealing with information that was missing or missed. With four experiments, we studied the completion of missing information despite full attention. Participants watched short soccer video clips and we informed participants that we removed a critical moment of ball contact in half of the clips. We asked participants to detect whether these moments of ball contact were present or absent. In Experiment 1, participants gave their detection responses either directly during an event or delayed after an event. Although participants directed their full attention toward the critical contact moment, they were more likely to indicate seeing the missing ball contact if it was followed by a causally matching scene than if it was followed by an unrelated scene, both for the immediate and delayed responses. Thus, event completion occurs quickly. In Experiment 2, only a causally matching scene but neither a white mask nor an irrelevant scene caused the completion of missing information. This indicates that the completion of missing information is caused by backward inferences rather than predictive perception. In Experiment 3, we showed that event completion occurs directly during a trial and does not depend on expectations built up after seeing the same causality condition multiple times. In Experiment 4, we linked our findings to event cognition by asking participants to perform a natural segmentation task. We conclude that observers complete missing information during coherent events based on a fast backward inference mechanism even when directing their attention toward the missing information.

## Significance

Both in their professional and private lives, people regularly make judgments on a sequence of causally connected events. Referees judge the course of potential foul play and eye-witnesses judge the course of an accident. However, observers sometimes judge to have seen something that was causally plausible but not present. A referee, for example, might believe to have seen a foul play following an interaction of two players that was never present just because one of the players falls to the ground. While the newly introduced video referees in sports mark a first step towards more fair play and more correct outcomes, it may not solve all problems. In this paper we argue that missing information within observed events is completed based on causal structures even when observers focus their attention on a specific action or movement. In particular, participants were more likely to indicate having seen the missing moment of ball interaction if it was followed by a causal continuation, such as a ball flying, than if it was followed by a non-causal continuation, such as players getting ready for a free kick. Importantly, filling in the missing moment of ball interaction occurred quickly. Further, this completion was the result of backward inferences (filling in the missing information based on the causal continuation) rather than predictive perception (anticipation of an upcoming ball interaction based on current motion information and expectations).

## Introduction

Human observers are surrounded by a complex, dynamic and rich world producing a vast amount of information that easily exceeds the available attentional resources and the capacity of working memory. Although observers regularly miss information, such as the moment of collision during a car crash or the contact between soccer players during a foul play, and although they can process only a limited amount of information concurrently, human observers usually perceive their environment as a succession of meaningful discrete events. These events guide observers’ comprehension, actions and memory in naturalistic environments (Bailey, Kurby, Giovannetti, & Zacks, 2013; Richmond, Gold, & Zacks, 2017; Swallow, Zacks, & Abrams, 2009). Event structure perception develops early in age (Wynn, 1996) and adults perceive event boundaries also without explicit instructions (Zacks et al., 2001). But how do observers deal with information that was missing or missed? In the situation of an observer not attending to a particular piece of information during perception, such as the moment of collision between two cars, it seems plausible that this information is filled in based on the causal consequence of an event, such as the position of the crashed cars. But is missing information also regularly filled in if observers attend to a specific piece of missing information, such as a video referee attending to the moment of body contact during a foul replay, or the participants in our experiment attending to the moment of ball contact during a shot? The answer to this question can provide insights into the distortions underlying judgments in professions in which observers assess causally related dynamic events, even when they are considered to process all necessary information with full attention.

We report a series of experiments studying this question. We investigated how participants fill in missing information during the perception of naturalistic events while relieving participants from the challenge of filtering the relevant information with their attention. That is, we explicitly instructed participants which information could be missing in the presented videos. Doing so, we were able to address the following questions: Does event completion occur immediately during the perception of an event (Experiment 1)? Is event completion caused by predictive perception or backward inferences (Experiments 2 and 3)? Is there a potential link between event completion and event segmentation (Experiment 4)?

### Completion of missing information

While observing and comprehending dynamic naturalistic scenes, observers need to deal with missing information regularly. Be it that information is missing perceptually, such as objects disappearing from view temporarily while moving behind an occluder, or be it that information is present but missed due to attentional lapses or due to looking or attending to other information.

On a perceptual level, there is a strong tendency of the visual system to complete missing information. If a circle is partly occluded by a square, for example, one still perceives the circle through amodal completion (Rauschenberger & Yantis, 2001). This strong tendency even involves the perception of illusory objects, such as the perception of the Kanizsa triangle through modal completion. The tendency to complete missing information occurs also in settings involving spatiotemporal information. If two circles appear sequentially at different locations, for example, the objects are not perceived as separate objects but as the same object linked by apparent motion as long as the objects stay within certain spatiotemporal boundaries (Larsen, Farrell, & Bundesen, 1983). This is also true for the tunnel effect (Burke, 1952) that describes the finding that observers perceive a single object passing through an occluder if they see an object moving toward the occluder that is reappearing from the other side of the occluder after a certain amount of time. Observers even perceive one object passing through the occluder if object features differ between entering and leaving the occluder (Burke, 1952).

Completion occurs not only on a basic perceptual level, but also for higher-level cognitive processes. In an experiment by Newtson and Engquist (1976), for example, participants saw short action sequences and were instructed to detect deletions of varying length during the film. Half of the deletions occurred during an event, and the other half of deletions occurred between events, that is at event boundaries. Whereas the number of detected deletions increased with increasing interval length at event boundaries, the number of detected deletions remained low independent of interval length during an event. Interpreting these findings from a completion standpoint, one could argue that coherent events are more resistant to deletions because they are combined into a coherent mental representation with deleted information being filled in. Strickland and Keil (2011) provided more direct evidence for such an interpretation with their report of the event-completion effect.

Event completion (Strickland & Keil, 2011) describes the finding that humans sometimes falsely remember that they have seen information that was missing within a causally coherent event. In their experiments, participants saw short action sequences such as a person approaching and kicking a ball. The films were composed of shots from different camera angles and the ball kick was followed by either a shot showing a causal continuation, such as a ball flying, or a shot showing a non-causal continuation, such as a person jogging. Importantly, the moment of ball contact was removed in half of the trials. After each clip, participants judged for a number of images whether they were present in the video clip or not. Critically, these pictures always included an image showing the moment of ball contact. Those participants, who actually did not see this image, were more likely to falsely report having seen the moment of ball contact when it was followed by a causal continuation compared to when it was followed by a non-causal continuation. Thus, observers complete missing information of naturalistic dynamic scenes based on their world knowledge of causal consequences.

Inspired by a controversial incident in the German Bundesliga where the ball entered the goal through a hole in the net on the side but the referee was sure that the ball had actually crossed the goal line, we studied the role of cognitive-perceptual expertise for the completion effect (Brockhoff, Huff, Maurer, & Papenmeier, 2016). We used the event-completion paradigm and showed participants with different soccer expertise (elite referees, players and novice) clips extracted from the footage of a real soccer game. Completion occurred for all three participant groups, suggesting that observers routinely complete missing events and that event completion arises independent of familiarity with stimuli or training.

One limitation of the original event-completion paradigm (Strickland & Keil, 2011) is that participants do not know what to look for. That is, participants are not aware of the manipulation and might not actually complete the missing information during the perception of the event but rather reason about the contact image during the test. With the second experiment conducted in our previous research (Brockhoff et al., 2016), we addressed this concern by telling participants about the manipulation upfront and asking them explicitly to indicate whether the moment of ball contact was present or absent after each trial. Despite the new task, participants still showed event completion as indicated by a lower contact-detection performance with causal continuation than with non-causal continuation. This demonstrates that event completion is not a testing artefact. However, our previous findings do not answer two fundamental questions: (1) How quickly does the event-completion effect occur during the observation of an event? and (2) which process causes the effect?

### Filling the gap: predictive perception vs. backward inferences

When filling in information that was missing during the perception of an event, such as reporting having seen a moment of ball contact that was missing in the perceptual stream, this filling in could result from at least two processes. On the one hand, observers might regularly generate predictions about upcoming perceptual information based on the currently perceived information. If some information is going to be missing in the perceptual stream, this information can be filled in by the predicted information. We will refer to this process as *predictive perception* in the following. On the other hand, observers might represent their perceived information in the form of mental models giving a description of the happenings in the perceptual world. Whenever new information is perceived, observers regularly try to map this incoming perceptual information to their existing mental model. If there is a gap between the state of the mental model and the information that is perceived, this gap is filled in during the mapping process and, thus, the contents of the gap are inferred based on the information following the missing information. We will refer to this process as *backward inferences* in the following. Whereas theories and research focusing on naturalistic visual events, such as the Event Segmentation Theory (EST) (Zacks, Speer, Swallow, Braver, & Reynolds, 2007) or predictive social perception (e.g., Bach & Schenke, 2017), emphasize the role of predictive perception during the comprehension of naturalistic events, theories and research on text processing (e.g., Graesser, Singer, & Trabasso, 1994; Haviland & Clark, 1974; Singer & Ferreira, 1983; Singer, Halldorson, Lear, & Andrusiak, 1992) emphasize the role of backward inferences during discourse comprehension. Thus, the role of both predictive perception and backward inferences during event comprehension is not yet clearly understood. In the following, we will give a short overview of both accounts.

Predictive perception is a central concept in the EST (Zacks et al., 2007). EST proposes that observers comprehend their dynamic environment based on event models stored in working memory. Those event models serve as stable representation of the perceptual world because they remain active as long as they closely resemble the happenings in the perceptual world. Predictive perception plays a crucial role in determining whether this still is the case. Thus, based on the current perceptual input and guided by the event model currently stored in working memory, observers constantly generate perceptual predictions about the near future. As long as those predictions closely resemble the actual perceptual input, the system remains in a stable state and the event model representation in working memory remains active. If an error detection mechanism detects a deviation between perceptual predictions and actual perceptual input that passes a certain threshold, the current event model is dismissed and a new event model based on the current perceptual input is generated in working memory. Thus, the mechanisms proposed by EST are both resistant to short periods of perceptual disruptions, such as occlusion or distraction, but also sensitive to major changes in the scenes, such as the beginning of a new event.

Research on predictive social perception investigating the processes involved during action observation proposes a prediction mechanism (e.g., Bach & Schenke, 2017; Hudson, Nicholson, Ellis, & Bach, 2016; Kilner, Friston, & Frith, 2007) that is functionally similar to EST. Based on assumptions about the external world – such as the football player is going to kick the ball – observers generate perceptual predictions that are then compared against the actual perceptual input. If there is a close enough match, missing information is filled in and ambiguous information is resolved based on the prior hypothesis. If there is a mismatch, the prior hypothesis and assumptions are revised. Thus, perceptual gaps should be filled in by the predicted perceptual input as long as those gaps are not followed by new semantic information that is not causally linked to observers’ prior assumptions.

Backward inferences, sometimes also called causal inferences or bridging inferences, are well studied in the context of text comprehension (e.g., Graesser et al., 1994; Haviland & Clark, 1974; Singer et al., 1992; Singer & Ferreira, 1983). When reading the sentences “Mary poured the water on the bonfire. The fire went out.” readers draw the inference that “the water extinguished the fire” (Singer et al., 1992). That is, the construction of coherent situation models during text comprehension involves drawing backward inferences (Schmalhofer, McDaniel, & Keefe, 2002; Zwaan & Radvansky, 1998). Given that the construction of situation models during text comprehension and the construction of event models during the comprehension of visual narratives both involve similar if not the same processes (Zacks, Speer, & Reynolds, 2009), backward inferences should also occur during event perception. Indeed, the existence of backward inferences during event perception was shown in recent studies involving picture stories (Magliano, Kopp, Higgs, & Rapp, 2017; Magliano, Larson, Higgs, & Loschky, 2016), demonstrating that missing bridging events are inferred during event perception. Thus, the filling in of perceptual gaps during event perception might not require any predictive processes at all. Instead, it is possible that the missing information is filled in based on the information following the perceptual gap using backward inferences. If causally coherent information follows the perceptual gap, these inferences can be drawn and the perceptual gap is filled in. If, however, the perceptual gap is followed by information that is not causally linked to the information prior to the perceptual gap, no backward inferences can be drawn and the gap cannot be filled in.

While a causal continuation is a prerequisite for backward inferences to occur, predictive perception accounts vary with respect to whether perceptual predictions also occur when no visual information is available. Whereas some accounts assume that perceptual predictions only become conscious (and thus result in perception) once they are confirmed by matching visual input (e.g., Enns & Lleras, 2008), other findings suggest that predictions are also active while the visual scene is occluded (Graf et al., 2007). The latter view is also supported by the results obtained with the representational momentum paradigm in which participants remember an object to be displaced into the direction of its motion once it is abruptly occluded by a mask (Freyd & Finke, 1984; Hubbard, 2005; Hudson et al., 2016). This occurs both under focused attention and is slightly increased with divided attention (Hayes & Freyd, 2002). In the following, we will refer to the latter view when discussing predictive perception: We assume that short perceptual gaps should be filled in based on perceptual predictions even in the absence of new semantic information confirming those predictions.

### Experimental overview

Our aim was to investigate the process underlying event completion in situations where observers’ full attention is deployed to the critical information, such as a video referee attending to the moment of body contact during a foul replay. In order to achieve this aim, we employed the contact-detection paradigm used in our previous research (Brockhoff et al., 2016) rather than the original memory based event-completion paradigm (Strickland & Keil, 2011) in our first three experiments. We fully debriefed our participants by instructing them which critical moment of ball contact would be missing in some of the video clips and asked them to detect whether it was missing or not. Whenever participants fill in the gap of missing information in the perceptual stream, they should report having seen something that was not present (high false-alarm rate) which leads to difficulties in distinguishing between information actually being present or absent in the perceptual stream (low contact-detection performance). With our experiments, we investigated whether event completion occurs quickly directly during event perception in such situations (Experiment 1), whether this completion is caused by predictive perception or backward inferences (Experiments 2 and 3), and whether event completion might be related to event perception (Experiment 4).

## Experiment 1

In our first experiment, we investigated whether observers complete missing information immediately while perceiving an event. We asked participants to either detect the critical moment of ball contact or ball release directly *during* event perception or to give their response *after* they finished watching the whole event. If completion occurs quickly, we should observe comparable completion effects both when giving responses directly during an event and when responses are delayed until after an event.

### Method

#### Participants

Sixty-four students of the University of Tübingen participated in the experiment in exchange for course credit. We determined the sample size using the following rule. In a previous experiment (Brockhoff et al., 2016), we had observed an effect size of *d* = 1.47 for the completion effect with delayed responses (dependent-measure sensitivity). In order to account for the fact that the effect size might be lower for immediate responses, we collected the data of 64 participants, which is large enough to detect effect sizes of *d* = .71 with a power of .8.

#### Apparatus and stimuli

**Fig. 1:**
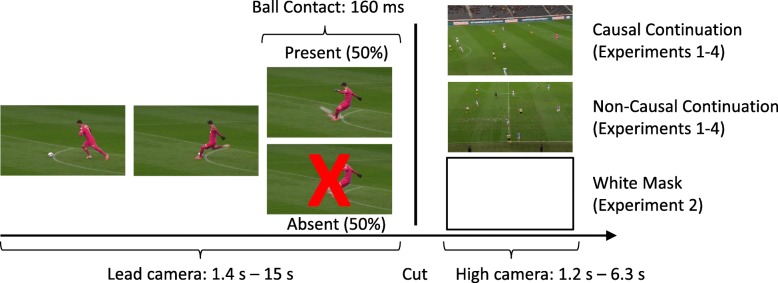
Illustration of the timing and stimulus variations used in our experiments. Participants’ task was to detect whether the critical moment of ball contact was either absent or present for each trial. We manipulated the continuation of the video clip after the cut. There was either a causal continuation, such as a ball flying across the field, a non-causal continuation, such as players preparing for a throw-in, or a white mask that covered the video content after the cut

We extracted short video clips from the video coverage of a soccer match between the Young Boys Bern and the Grasshoppers Zürich that took place on 23 March 2014 as stimulus material. We created the video clips according to the following rules (see Fig. 1). Each video clip consisted of two parts combined by a filmic cut. The first part of each clip was extracted from the footage of the lead camera focusing on the player in ball possession and depicting this player including some of its surrounding (duration: 1.4 to 15 s). The second part of each clip was extracted from the footage of the high camera showing a larger part of the soccer field (duration: 1.2 to 6.3 s). The end of the first part of each clip depicted a clear action of a player toward the ball: 14 × kick-off, 5 × corner kick, 13 × throw-in, 8 × free kick. In the complete conditions, the moment of ball contact (e.g., player hitting the ball for kick-off) or ball release (e.g., ball just released from the player’s hand at throw-ins) occurred in the third last frame of the first part of the clip (presentation rate: 25 frames per second). In the removed conditions, we deleted the last four frames (160 ms) of the first part of the clips, thus resulting in the critical moment of ball contact or ball release not being visible anymore. In half of the stimuli, the second part of each clip depicted a causal continuation of the first part of each clip, such as a ball flying. In the other half of stimuli, the second part of each clip depicted a non-causal continuation, such as a player injury or the players getting ready for a free kick. In order to increase the readability of this article, we will refer to the critical moment of ball interaction as contact moment in the remainder of this article, irrespective whether it actually was a ball contact, such as a kick, or a ball release, such as a throw-in. Note that we ensured that the critical moment of ball contact was never visible following the cut.

Participants were placed at an unrestricted viewing distance of approximately 60 cm to the display. We presented the video clips on a gray background using PsychoPy (Peirce, 2007, 2009). The size of the video clips was 31.5° of visual angle horizontally and 18.1° of visual angle vertically.

#### Procedure and design

At the beginning of the experiment, participants gave informed consent. Thereafter, they performed two versions of the experiment with order of the experimental versions balanced across participants. In the immediate-response version of the experiment, we instructed participants to press the spacebar immediately after detecting the critical moment of ball contact. We analyzed the first response following the contact moment. In the delayed-response version of the experiment, we instructed participants to indicate whether they had seen the critical moment of ball contact after each trial, using a rating scale ranging from 1 (sure no) to 6 (sure yes). Our instruction explaining the task to the participants contained drawings of a player performing a throw-in and just releasing the ball as well as of a player kicking a ball and just touching the ball with the toe of the shoe in order to make sure that participants understood what we meant by the critical moment of ball contact. Each experimental version consisted of 40 different clips (trials) with trial order randomized for each participant. The association of clips to conditions was balanced across participants, that is, each clip occurred equally often within each condition across all participant. Participants were allowed to take self-paced breaks between trials.

Each participant saw only one causality condition. Further, we manipulated the presence of the contact moment within subjects. This resulted in a 2 (causality: causal, non-causal; between) × 2 (contact: present, absent; within) design for both experimental versions (immediate response, delayed response). For each experimental version, there were 20 repetitions per condition for each participant. There were no practice trials.

### Results

We calculated the dependent-measure sensitivity (d’) as an indicator of contact-detection performance and we calculated the dependent-measure response criterion (c) as an indicator of response bias for both experimental versions. Because d’ and c are not defined for hit rates and false-alarm rates of 1.0 or 0.0, we replaced these values by half a trial incorrect or half a trial correct, respectively.

#### Immediate response

**Fig. 2:**
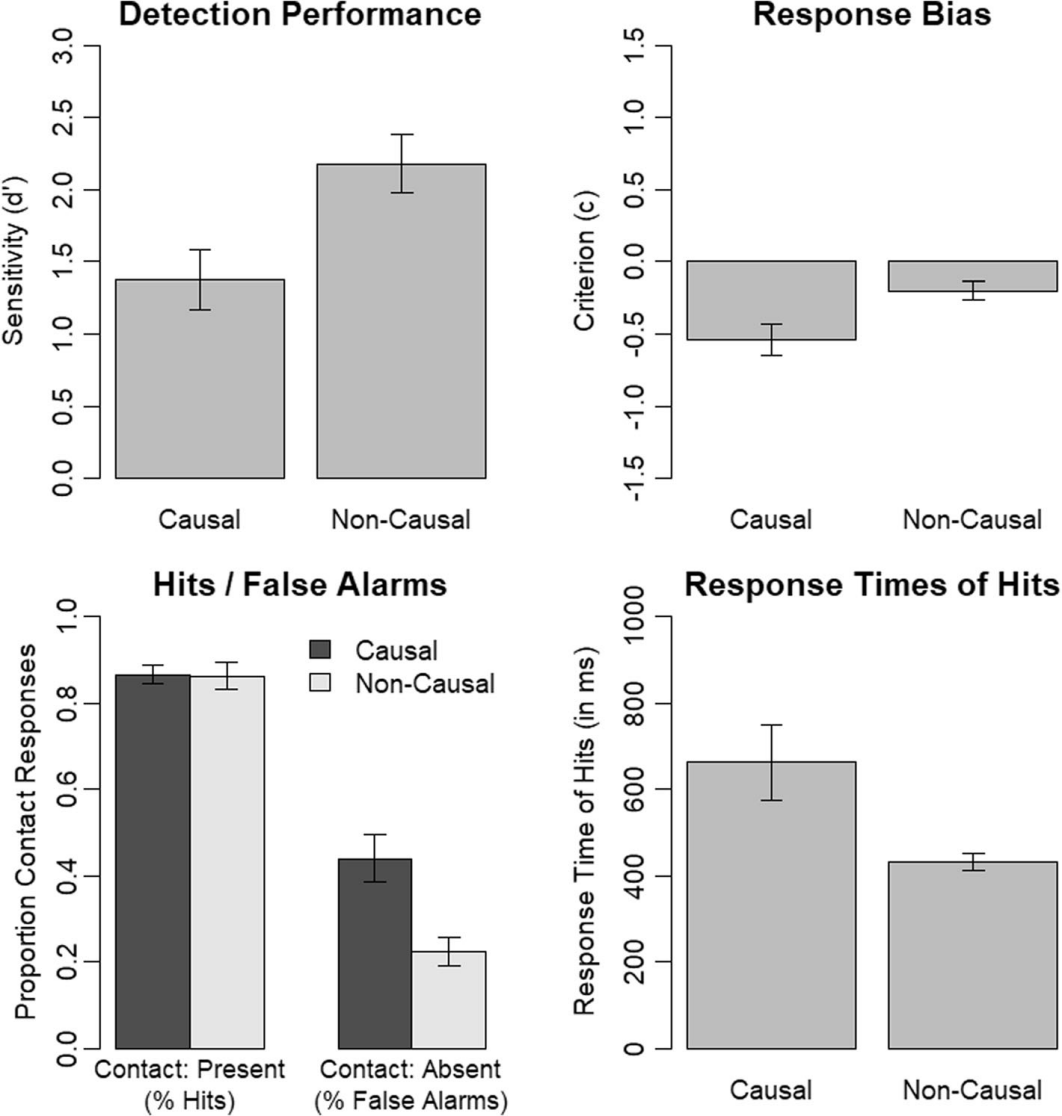
Results of Experiment 1 (immediate-response experimental version). Error bars indicate the standard error of the mean

Results obtained in the immediate-response experimental version are depicted in Fig. 2. We observed a lower contact-detection performance for participants watching the video clips that presented a causal continuation compared to participants watching the video clips that had a non-causal continuation, *t*(62) = − 2.77, *p* = .007. Furthermore, participants’ response bias was more liberal (more contact responses) when viewing causal continuations than non-causal continuations, *t*(50.17) = − 2.66, *p* = .011 (Welch’s unequal variances *t* test). We further investigated this result pattern with a mixed analysis of variance (ANOVA) containing the factors contact (present, absent; within) and causality (causal, non-causal; between) and the dependent-measure proportion of contact responses. There was a significant interaction of contact and causality, *F*(1, 62) = 7.23, *p* = .009, *η*_*p*_^2^ = .10. We used follow-up Welch’s unequal variances *t* tests to further investigate this interaction. This revealed that the reduced contact-detection performance was the result of an event-completion effect, that is, the presence of a causal continuation instead of a non-causal continuation resulted in an increased false-alarm rate, *t*(50.00) = 3.35, *p* = .002, but in no change of the hit rate, *t*(54.51) = 0.08, *p* = .935. Thus, participants showed a higher tendency to falsely report having seen the contact moment although it was not present if they saw a causal continuation instead of a non-causal continuation. The other effects of the ANOVA were as follows. There was a significant main effect of contact, *F*(1, 62) = 180.63, *p* < .001, *η*_*p*_^2^ = .74, and a significant main effect of causality, *F*(1, 62) = 9.67, *p* = .003, *η*_*p*_^2^ = .13.

We analyzed response times of hits using a Welch’s unequal variances *t* test. Participants response times in the causal condition were slower than response times in the non-causal condition, *t*(34.28) = 2.60, *p* = .014. This pattern of results goes in line with the reduced contact-detection performance in the causal condition. Interestingly, the mean response times in the causal condition (662 ms) indicate that event completion occurs quickly.

#### Delayed response

**Fig. 3:**
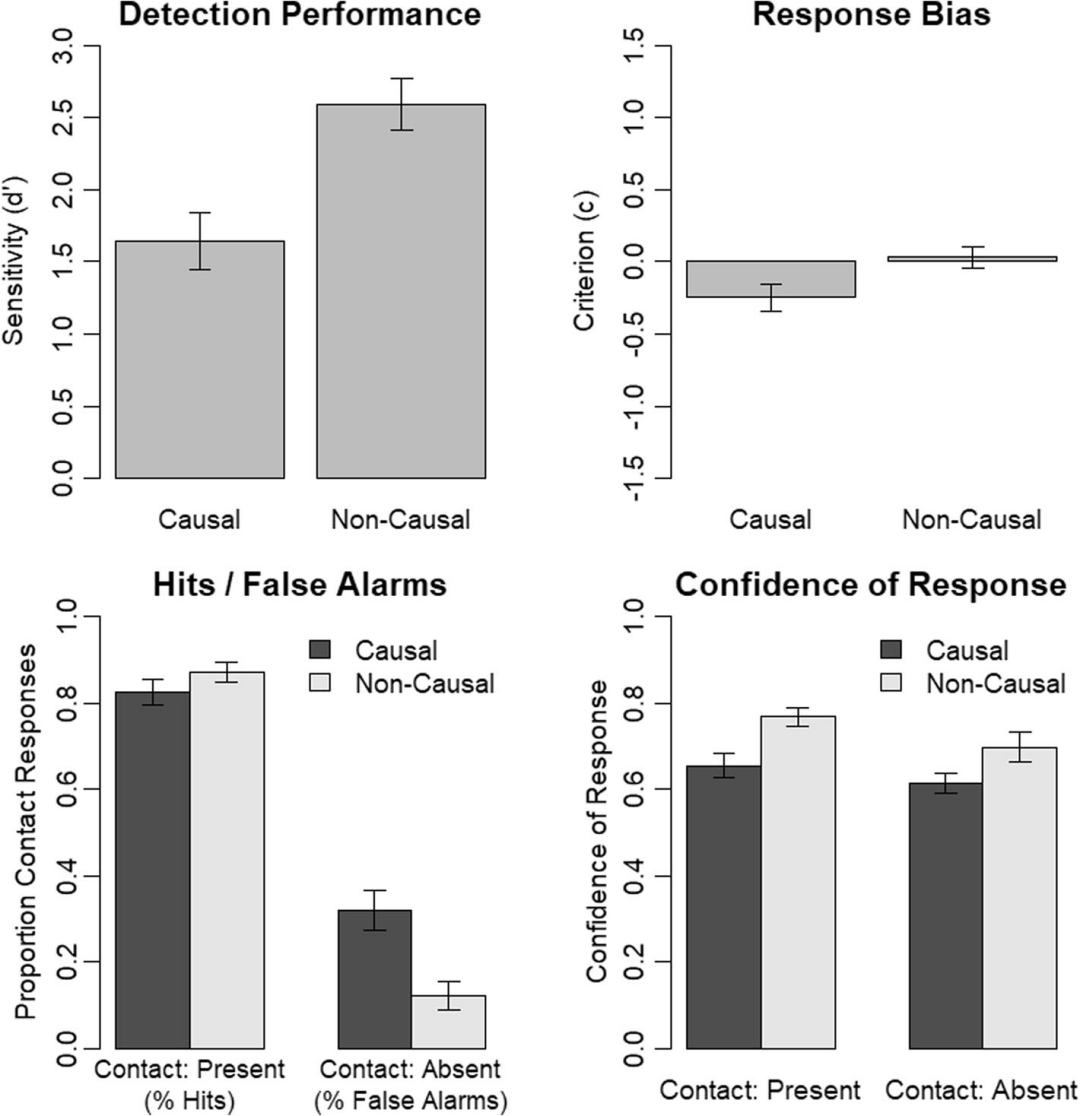
Results of Experiment 1 (delayed-response experimental version). Error bars indicate the standard error of the mean

Results obtained in the delayed-response experimental version are depicted in Fig. 3. Based on participants’ responses to the rating scale, we obtained measures of contact-present responses, contact-absent responses as well as a measure of confidence of response. We treated responses of one to three as contact-absent responses and responses of four to six as contact-present responses. Confidence was 0 for responses three and four, 0.5 for responses two and five, and 1.0 for responses one and six.

We observed a lower contact-detection performance and a more liberal response criterion in the causal than non-causal condition, *t*(62) = − 3.57, *p* = .001 and *t*(62) = − 2.30, *p* = .025, respectively. A mixed ANOVA containing the factors contact (present, absent; within) and causality (causal, non-causal; between) and the dependent-measure proportion of contact responses revealed a significant interaction of contact and causality, *F*(1, 62) = 11.04, *p* = .002, *η*_*p*_^2^ = .15. This was caused by an increased false-alarm rate in the causal compared with the non-causal condition, *t*(62) = 3.44, *p* = .001. There was no significant difference in hit rates across the causality conditions, *t*(62) = − 1.21, *p* = .231. Thus, we also observed an event-completion effect in the delayed-response experimental version. The other effects of the ANOVA were as follows. There was a significant main effect of contact, *F*(1, 62) = 294.77, *p* < .001, *η*_*p*_^2^ = .83, and a significant main effect of causality, *F*(1, 62) = 5.69, *p* = .020, *η*_*p*_^2^ = .08.

We analyzed the confidence of responses using a mixed ANOVA containing the factors contact (present, absent; within) and causality (causal, non-causal; between). There was a significant main effect of causality, *F*(1, 62) = 9.42, *p* = .003, *η*_*p*_^2^ = .13, that is, participants were more confident in their responses in the non-causal condition in which they also showed a higher contact-detection performance. The main effect of contact was also significant, *F*(1, 62) = 6.37, *p* = .014, *η*_*p*_^2^ = .09, that is, participants were more confident in their responses if the contact moment was present than if the contact moment was absent. Importantly, however, the interaction of causality and contact was not significant, *F*(1, 62) = 0.46, *p* = .502, *η*_*p*_^2^ = .01. Thus, we did not find any evidence of participants being less confident in the condition without contact moment and with causal continuation. That is, although participants’ tendency to false alarm selectively increased in this condition, they were not selectively less confident in their responses.

#### Comparison: Immediate response vs. delayed response

We ran a comparison across the two experimental versions in order to investigate whether event completion differs between participants responding immediately after the contact moment and participants responding at the end of each clip. First, we compared contact-detection performance across experimental versions using a mixed ANOVA containing the factors causality (causal, non-causal; between) and experiment (immediate, delayed; within) and the dependent-measure sensitivity (d’). There was a significant main effect of causality, *F*(1, 62) = 13.44, *p* = .001, *η*_*p*_^2^ = .18, indicating a reduced contact-detection performance in the causal condition as compared with the non-causal condition. The main effect of experiment was also significant, *F*(1, 62) = 5.80, *p* = .019, *η*_*p*_^2^ = .09, indicating a higher contact-detection performance in the delayed-response experimental version than the immediate-response experimental version. Importantly, however, the interaction of causality and experiment was not significant, *F*(1, 62) = 0.26, *p* = .613, *η*_*p*_^2^ < .01. Thus, the effect of causality on contact-detection performance was not influenced by the experimental version.

Second, we compared response bias across experimental versions using a mixed ANOVA containing the factors causality (causal, non-causal; between) and experiment (immediate, delayed; within) and the dependent-measure response criterion (c). There was a significant main effect of causality, *F*(1, 62) = 10.31, *p* = .002, *η*_*p*_^2^ = .14, indicating a more liberal response bias in the causal condition as compared with the non-causal condition. The main effect of experiment was also significant, *F*(1, 62) = 10.95, *p* = .002, *η*_*p*_^2^ = .15, indicating a more liberal response bias in the immediate-response experimental version than the delayed-response experimental version. Importantly, however, the interaction of causality and experiment was again not significant, *F*(1, 62) = 0.14, *p* = .706, *η*_*p*_^2^ < .01. Thus, the effect of causality on response bias was also not influenced by the experimental version.

We also investigated the influence of experimental version on the hit rates and false-alarm rates using a mixed ANOVA containing the factors causality (causal, non-causal; between), contact (present, absent; within) and experiment (immediate, delayed; within) and the dependent-measure proportion of contact responses. Again, the event-completion effect did not differ across experimental version as indicated by a non-significant three-way interaction of causality, contact and experiment, *F*(1, 62) = 0.15, *p* = .699, *η*_*p*_^2^ < .01. The other effects of the ANOVA were as follows. The significant main effects of causality, *F*(1, 62) = 13.34, *p* = .001, *η*_*p*_^2^ = .18, and contact, *F*(1, 62) = 309.62, *p* < .001, *η*_*p*_^2^ = .83, as well as the significant interaction of causality and contact, *F*(1, 62) = 11.95, *p* = .001, *η*_*p*_^2^ = .16, correspond to the findings that we reported above in the respective individual ANOVAs of the immediate-response experimental version and delayed-response experimental version. Further, there was a significant main effect of experiment, *F*(1, 62) = 8.43, *p* = .005, *η*_*p*_^2^ = .12, indicating a slightly higher proportion of contact responses in the immediate-response experimental version than delayed-response experimental version. The interaction of contact and experiment was also significant, *F*(1, 62) = 6.13, *p* = .016, *η*_*p*_^2^ = .09, corresponding to the higher contact-detection performance in the delayed-response experimental version than in the immediate-response experimental version. The interaction of causality and experiment was not significant, *F*(1, 62) = 0.59, *p* = .447, *η*_*p*_^2^ = .01.

## Experiment 2

With Experiment 1, we showed that event-completion occurs quickly during the perception of an event. It remains still unresolved, however, as to whether event completion is caused by predictive perception or backward inferences. With the present experiment, we investigated this question by replacing the second part of the video clip with a white mask in half of the trials. Backward inferences are drawn based on the information following the perceptual gap. Thus, backward inferences can only be drawn if the missing information is followed by a causal continuation but not if it is followed by a white mask. Accordingly, if event completion is caused by backward inferences, we should observe completion only in the condition with causal continuation and no mask. In contrast, perceptual predictions are generated based on the information that is present before the perceptual gap. Importantly, we assume that those predictions should not be disrupted by perceptual interruptions such as a mask, similar to the representational momentum paradigm (Freyd & Finke, 1984; Hubbard, 2005; Hudson et al., 2016). Instead, perceptual prediction should only be disrupted by the presence of new semantic information that does not match those perceptual predictions. Accordingly, if event completion is caused by predictive perception, we should observe completion in all conditions with a white mask as well as in the condition with causal continuation and no mask. Only in the condition with no causal continuation and no mask, event completion should not occur.

### Method

#### Participants

Forty students from the University of Tübingen participated in this experiment in exchange for course credit or monetary compensation. We determined the sample size based on a power analysis with the R package powerbydesign (Papenmeier, 2018). As an estimator for the expected sensitivity values, we used the values obtained in the immediate-response experimental version of Experiment 1. The power analysis revealed that we required a sample size of at least 38 in order to achieve a power of .8 for our predicted interaction. Because we required a sample size that can be divided by four in order to counter-balance our video clips across conditions and participants, we collected the data of 40 participants.

#### Apparatus and stimuli

We used the same apparatus and stimuli as in Experiment 1. In addition, we created new “mask” stimuli. For these stimuli, we used the stimuli of Experiment 1 and replaced the second part of each clip with a white mask (see Fig. 1). Importantly, the duration of the mask was the same as the duration of the second part of the original clips. That is, the duration of the clips was the same in the mask and no-mask conditions.

#### Procedure and design

We used the same procedure as in the immediate-response experimental version of Experiment 1. In contrast to Experiment 1, we added the new factor mask (mask, no-mask; within-subjects) to the design. Thus, the second part of each clip showed a semantic continuation (causal vs. non-causal; between-subjects) for half of the trials and a white mask for the other half of trials. This resulted in a 2 (causality: causal, non-causal; between) × 2 (contact: present, absent; within) × 2 (mask: mask, no mask; within) design. There were 20 repetitions per condition for each participant. There were no practice trials. Participants saw each clip twice, once with and once without mask, thus completing 80 experimental trials. Trial order was randomized for each participant.

### Results

**Fig. 4:**
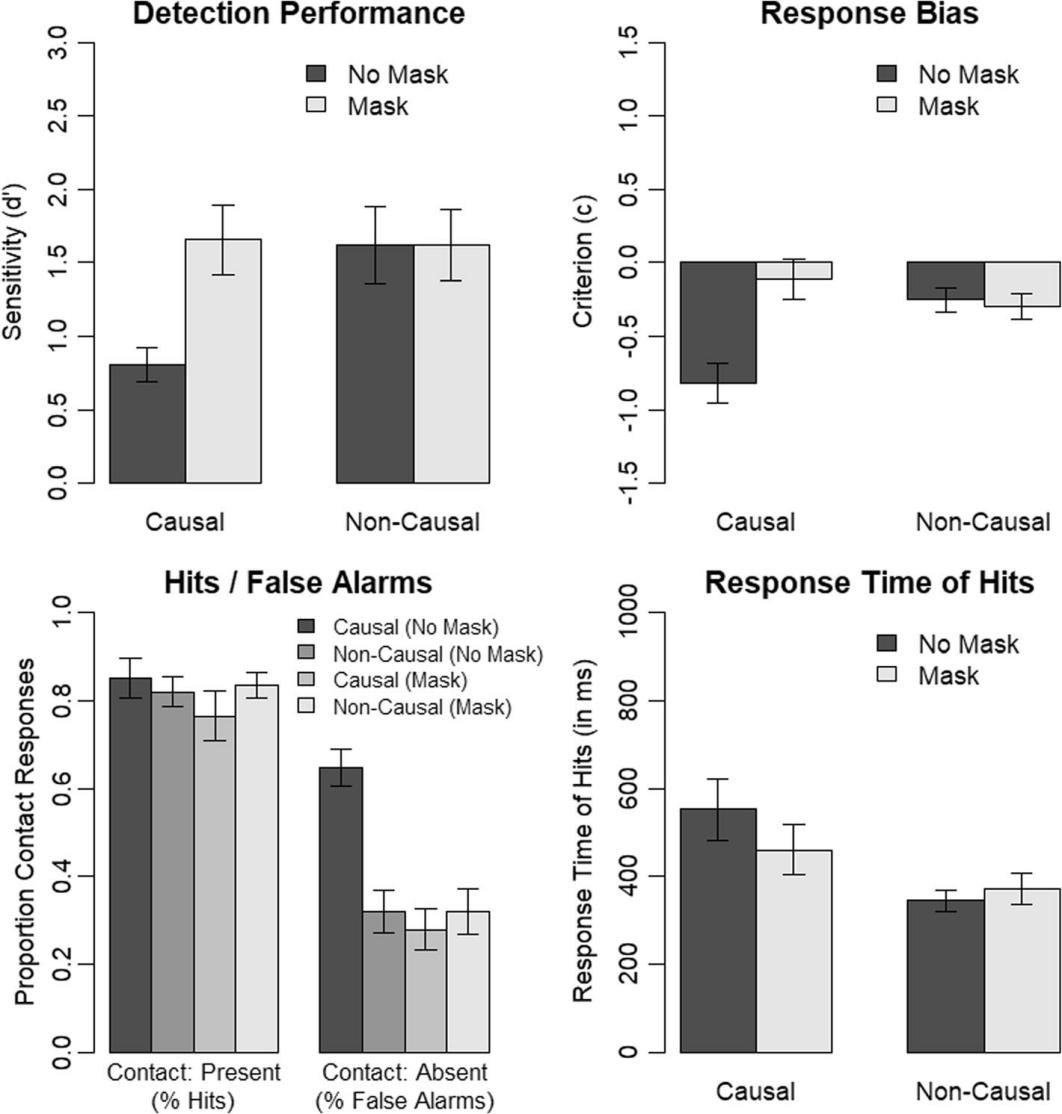
Results of Experiment 2. Error bars indicate the standard error of the mean

We analyzed contact-detection performance using the dependent-measure sensitivity (d’) and response bias using the dependent-measure response criterion (c). Because d’ and c are not defined for hit rates and false-alarm rates of 1.0 or 0.0, we replaced these values by half a trial incorrect or half a trial correct respectively. We calculated a mixed ANOVA using the factors causality (causal, non-causal; between) and mask (mask, no mask; within) and the dependent measure d’ (see Fig. 4). Most importantly, there was a significant interaction of causality and mask, *F* (1, 38) = 13.97, *p* = .001, *η*_*p*_^2^ = .27. We investigated the interaction using follow-up paired *t* tests. For participants assigned to the non-causal condition, there was no significant difference in contact-detection performance across mask conditions, *t* (19) = 0.01, *p* = .993. For participants assigned to the causal condition, however, contact detection was lower when they saw a causal continuation instead of white mask after the cut, *t* (19) = − 4.47, *p* < .001. This indicates that a causal continuation is necessary for event completion to occur. The other effects of the ANOVA were as follows. The main effect of mask was significant, *F* (1, 38) = 13.89, *p* = .001, *η*_*p*_^2^ = .27, and the main effect of causality was not significant, *F* (1, 38) = 1.78, *p* = .190, *η*_*p*_^2^ = .04.

For the response bias, we observed results comparable to contact-detection performance. We calculated a mixed ANOVA using the factors causality (causal, non-causal; between) and mask (mask, no mask; within) and the dependent measure c (see Fig. 4). Most importantly, there was also a significant interaction of causality and mask, *F* (1, 38) = 16.45, *p* < .001, *η*_*p*_^2^ = .30. We investigated the interaction using follow-up paired *t* tests. For participants assigned to the non-causal condition, there was no significant difference in response bias across mask conditions, *t* (19) = 0.43, *p* = .669. For participants assigned to the causal condition, however, the response bias was more liberal when they saw a causal continuation instead of white mask after the cut, *t* (19) = − 4.54, *p* < .001. This further supports our interpretation based on contact-detection performance that a causal continuation is necessary for event completion to occur. The other effects of the ANOVA were as follows. The main effect of mask was significant, *F* (1, 38) = 12.86, *p* = .001, *η*_*p*_^2^ = .25, and the main effect of causality was not significant, *F* (1, 38) = 2.20, *p* = .146, *η*_*p*_^2^ = .05.

As in Experiment 1, we ran an additional analysis on the proportion of contact responses to investigate the influence of our manipulations on hits and false alarms. Thus, we calculated a mixed ANOVA with the factors contact (present, absent; within), causality (causal, non-causal; between) and mask (mask, no mask; within) and the dependent-measure proportion of contact responses. Most importantly, there was a significant three-way interaction of contact, causality and mask, *F* (1, 38) = 14.72, *p* < .001, *η*_*p*_^2^ = .28. As is evident from Fig. 4, this interaction is mainly driven by a selective increase in false-alarm rates in the condition with causal continuation and no mask. This supports our conclusion from the contact-detection performance analysis, that event completion occurred only when a causal continuation was shown and no mask was present. In contrast, the masked conditions and non-causal continuation conditions caused similar response patterns. This pattern of results suggests that event completion is caused by backward inferences rather than predictive perception. The other effects of the ANOVA were as follows. There was a non-significant main effect of causality, *F* (1, 38) = 2.86, *p* = .099, *η*_*p*_^2^ = .07, a significant main effect of mask, *F* (1, 38) = 16.58, *p* < .001, *η*_*p*_^2^ = .30, a significant main effect of contact, *F* (1, 38) = 108.30, *p* < .001, *η*_*p*_^2^ = .74, a significant interaction of causality and mask, *F* (1, 38) = 18.93, *p* < .001, *η*_*p*_^2^ = .33, a non-significant interaction of causality and contact, *F* (1, 38) = 4.01, *p* = .052, *η*_*p*_^2^ = .10, and a significant interaction of mask and contact, *F* (1, 38) = 18.21, *p* < .001, *η*_*p*_^2^ = .32.

In a final analysis, we investigated response times for hits. We calculated a mixed ANOVA using the factors causality (causal, non-causal; between) and mask (mask, no mask; within) and the dependent-measure response times for hits. There was a significant main effect of causality, *F* (1, 38) = 6.56, *p* = .015, *η*_*p*_^2^ = .15. The main effect of mask, *F* (1, 38) = 0.66, *p* = .420, *η*_*p*_^2^ = .02, and the interaction of causality and mask, *F* (1, 38) = 2.28, *p* = .139, *η*_*p*_^2^ = .06, were not significant. Thus, there was a general increase of response times for participants assigned to the causal condition. As was the case in Experiment 1, mean response times in the causal continuation without mask condition indicate that event completion occurs quickly.

## Experiment 3

One potential concern regarding our previous experiments is the fact that we manipulated causality between subjects. Thus, it remains possible that it was not the causal continuation within a single video clip that caused the event-completion effect based on backward inferences. Rather, participants might have generated expectations about the causal structure of the video clips across repeated presentations of either causal or non-causal continuations. In order to investigate this idea, we ran the following control experiment. We manipulated causality within-subjects, that is participants saw both causal and non-causal continuations throughout the experiment. Further, we manipulated the order of trials. We either presented all trials randomly intermixed, or we presented the causal and non-causal trials in two separate blocks. If event completion is caused by backward inferences based on the causal continuation within a single trial, we should observe a comparable event-completion effect across both types of trial-order (intermixed, blocked). If expectations about the causal structure of the video clips influence event completion, however, the event-completion effect should be stronger with a blocked trial order than an intermixed trial order.

### Method

#### Participants

Our sample consisted of 64 students from the University of Tübingen. They participated in this experiment in exchange for course credit or monetary compensation. One participant did not do the task (did not detect a single contact moment across the whole experiment) and was thus removed from the sample and replaced by a new participant. We determined the sample size based on a power analysis with the R package powerbydesign (Papenmeier, 2018). As an estimator for the expected sensitivity values, we used the values obtained in the immediate-response experimental version of Experiment 1. The power analysis revealed that we required a sample size of at least 58 in order to achieve a power of at least .8 for each of the two hypothesized outcomes, that is either the interaction of trial order and causality or a main effect of causality only. Because we required a sample size that can be divided by eight in order to counter-balance our video clips across conditions and participants, we collected the data of 64 participants.

#### Apparatus and stimuli

We used the same apparatus and stimuli as in Experiment 1.

#### Procedure and design

We used the procedure of the immediate-response experimental version of Experiment 1 with the following changes. Causality was manipulated within subjects instead of between subjects. Furthermore, we varied the trial order of the causality trials. Participants either saw all trials in an intermixed order or they saw two blocks of trials with the causal conditions in one block and the non-causal conditions in the other block (block order counter-balanced across respective participants). Besides the blocking of causality for half of the participants, trials were presented in a randomized order for each participant. The assignment of video clips to conditions was again balanced across participants, ensuring that each video clip occurred equally often in each condition across all participants. Our changes resulted in a 2 (causality: causal, non-causal; within) × 2 (contact: present, absent; within) × 2 (trial order: blocked, intermixed; between) design. There were 10 repetitions per condition for each participant. There were no practice trials.

### Results

We analyzed contact-detection performance using the dependent-measure sensitivity (d’) and response bias using the dependent-measure response criterion (c). Because d’ and c are not defined for hit rates and false-alarm rates of 1.0 or 0.0, we replaced these values by half a trial incorrect or half a trial correct, respectively.

**Fig. 5:**
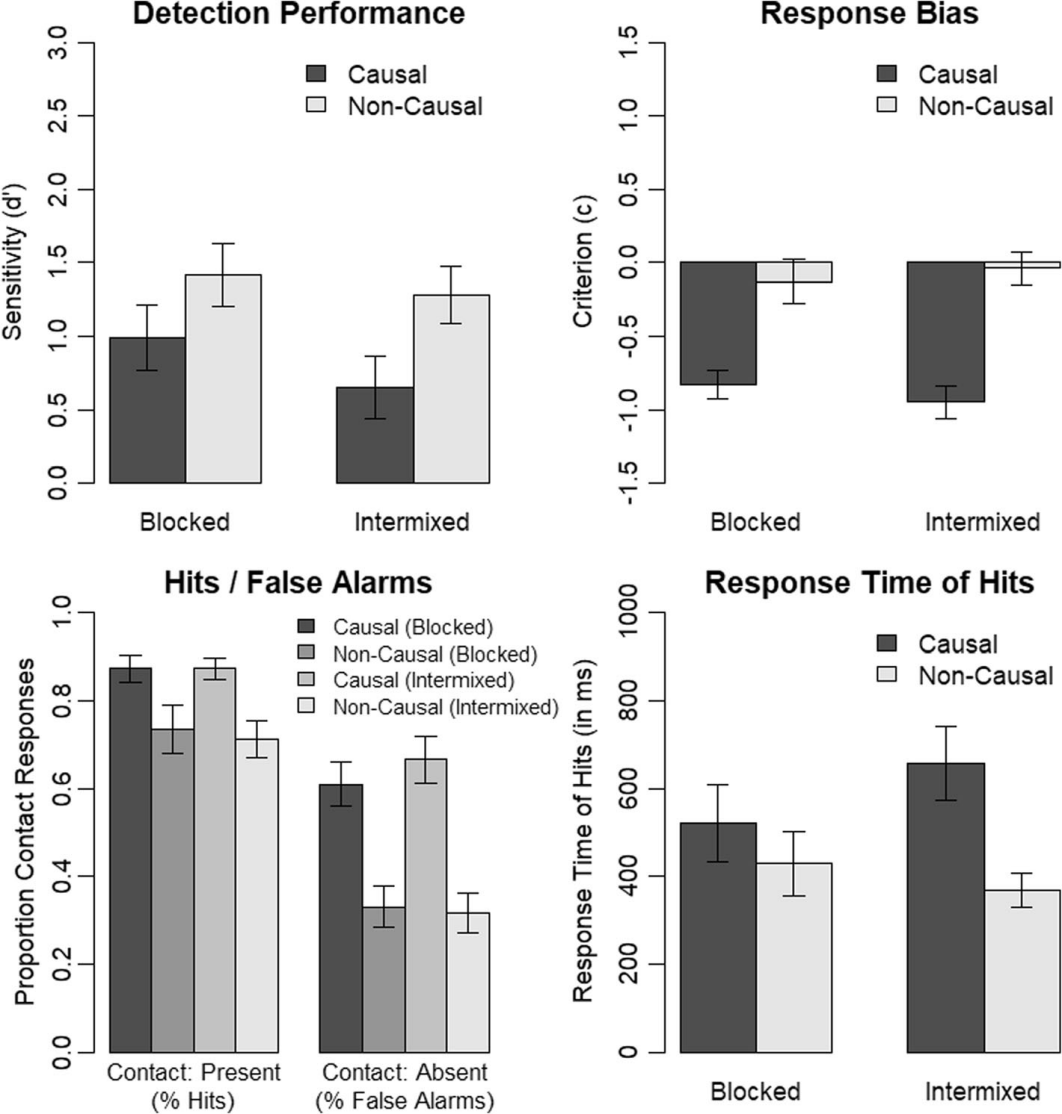
Results of Experiment 3. Error bars indicate the standard error of the mean

We analyzed the dependent measure d’ (see Fig. 5) using a mixed ANOVA with the factors causality (causal, non-causal; within) and trial order (blocked, intermixed; between). We replicated the significant effect of causality on contact change detection performance, *F*(1, 62) = 13.88, *p* < .001, *η*_*p*_^2^ = .18. Contact-detection performance was reduced in the causal as compared with the non-causal condition. Importantly, there was no significant interaction of causality and trial order, *F*(1, 62) = 0.53, *p* = .470, *η*_*p*_^2^ = .01. That is, event completion occurred both with an intermixed trial order and a blocked trial order. We can, thus, conclude that event completion occurred directly during the perception of the video clips in our experiments and that it did not rely on expectations that participants built up only after repeated occurrences of the same causality condition. There was no significant main effect of trial order, *F*(1, 62) = 0.84, *p* = .364, *η*_*p*_^2^ = .01.

We analyzed response bias using a mixed ANOVA with the factors causality (causal, non-causal; within) and trial order (blocked, intermixed; between). There was a significant main effect of causality, *F*(1, 62) = 56.03, *p* < .001, *η*_*p*_^2^ = .47, but neither a significant main effect of trial order, *F*(1, 62) = 0.01, *p* = .906, *η*_*p*_^2^ < .01, nor a significant interaction of causality and trial order, *F*(1, 62) = 0.98, *p* = .327, *η*_*p*_^2^ = .02. This further supports our interpretation that event completion occurs also with an intermixed trial order and is, thus, not dependent on expectations that are built up only after repeated occurrences of the same causality condition.

We ran an additional analysis on the proportion of contact responses to investigate the influence of our manipulations on hits and false alarms. Thus, we calculated a mixed ANOVA with the factors contact (present, absent; within), causality (causal, non-causal; within) and trial-order (blocked, intermixed; between) and the dependent-measure proportion of contact responses. Again, our pattern of results was not influenced by trial order as indicated by a non-significant main effect of trial order, *F*(1, 62) = 0.02, *p* = .886, *η*_*p*_^2^ < .01, a non-significant interaction of trial order and contact, *F*(1, 62) = 0.21, *p* = .651, *η*_*p*_^2^ < .01, a non-significant interaction of trial order and causality, *F*(1, 62) = 0.55, *p* = .462, *η*_*p*_^2^ = .01, as well as a non-significant three-way interaction of trial order, contact and causality, *F*(1, 62) = 0.42, *p* = .518, *η*_*p*_^2^ = .01. Instead, we replicated the result pattern of our previous experiments showing a significant two-way interaction of contact and causality, *F*(1, 62) = 20.73, *p* < .001, *η*_*p*_^2^ = .25, a significant main effect of contact, *F*(1, 62) = 76.94, *p* < .001, *η*_*p*_^2^ = .55, and a significant main effect of causality, *F*(1, 62) = 56.58, *p* < .001, *η*_*p*_^2^ = .48. That is, we again observed an increase in false-alarm rates in conditions with a causal rather than non-causal continuation. In contrast to our previous experiments, however, there was also a decrease in hit rates in conditions with no causal continuation as compared to conditions with causal continuation.

In a final analysis, we investigated response times for hits. We calculated a mixed ANOVA using the factors causality (causal, non-causal; within) and trial order (blocked, intermixed; between) and the dependent-measure response times for hits. We included only participants with at least two hits per condition into this analysis in order to ensure reliable response times estimates for each participant. Thus, we removed four participants from the data set prior to this analysis. There was a significant main effect of causality, *F*(1, 58) = 14.72, *p* < .001, *η*_*p*_^2^ = .20, replicating the results of our previous experiments that participants show longer response times for hits in trials showing a causal continuation than in trials showing a non-causal continuation. Furthermore, both the main effect of trial order, *F*(1, 58) = 0.17, *p* = .677, *η*_*p*_^2^ < .01, and the interaction of trial order and causality, *F*(1, 58) = 3.79, *p* = .056, *η*_*p*_^2^ = .06, were not significant. This indicates that trial order did not modulate the effect of causality on response times for hits in our experiment. Nonetheless, it is worth noting that the two-way interaction was close to significant, indicating that the effect of causality on response times for hits might be somewhat stronger with an intermixed trial order than a blocked trial order. Most important to the aim of the present experiment, however, the response time effect was clearly evident also with an intermixed trial order indicating that it was also not a result of expectancies built up during repeated presentations of the same causality condition.

## Experiment 4

With our Experiments 1–3 we demonstrated that event completion occurs quickly during the perception of events and that the perceptual gaps are filled in by backward inferences. This indicates that event completion occurs as a result of the construction of coherent event models during event perception. Because the construction of mental models during event perception is supported by both backward inferences and event segmentation (Magliano, Loschky, Clinton, & Larson, 2013), we conducted a final experiment asking participants to perform an event segmentation task with the stimuli used in our event-completion experiments. We hypothesized that the presence of a causal continuation should lead to a reduced segmentation behavior as compared with the presence of a non-causal continuation. This would indicate that participants perceive a more coherent event. Importantly, this should also be true for the video clips where we deleted four frames, providing converging evidence that participants fill in the perceptual gap resulting in the perception of coherent events. As control stimuli, we also presented clips where we removed even more frames before the cut. This should make the causal link less salient, thus reducing the effect of causal continuation on event segmentation.

### Method

#### Participants

Forty students from the University of Tübingen participated in this experiment in exchange for course credit or monetary compensation.

#### Apparatus and stimuli

We used the same apparatus as in Experiment 1. In addition to the stimuli from Experiment 1, we created new stimuli for which we removed nine frames instead of four frames. Thus, we had three versions of each clip in this experiment: contact present, four frames removed and nine frames removed.

#### Procedure and design

Participants performed an event segmentation task in this experiment. After participants gave informed consent, we instructed them to press a button whenever they perceived that a natural and meaningful segment had ended. We informed participants that there were no right or wrong answers but that we were interested in their perception of meaningful segments. Participants performed the natural event segmentation task for all 40 video clips. There was a fixed 5-s break between the video clips. Participants saw half of the trials with contact present and the other half of trials with contact removed. Of the contact removed trials, half of the trials belonged to the four frames removed condition and half of the trials belonged to the nine frames removed condition. As in the previous experiments, we manipulated causality between subjects, that is half of the participants saw a causal continuation in all video clips and the half of participants saw a non-causal continuation in all video clips. The association of video clips to conditions was counter-balanced across participants, that is, each clip occurred equally often in each condition across participants.

Our manipulations resulted in a 2 (causality: causal, non-causal; between) × 3 (contact: present, four frames removed, nine frames removed; within) design. There were no practice trials. Trial order was randomized for each participant.

### Results

We analyzed the proportion of segmentation responses in the first second following the cut of each video clip.^1^ We choose this time interval for two reasons. First, our manipulations were introduced using the cut and we were interested in the segmentation responses associated with our manipulations. Thus, it is natural to use the cut as the beginning of the time interval used for this analysis. Second, the content following each cut differed between the causal and non-causal conditions by definition. Thus, we restricted the time interval to the first second following the cut in order to ensure that we only looked at segmentation responses associated with our manipulations and not with any follow-up events present in the later content of the clips.

**Fig. 6:**
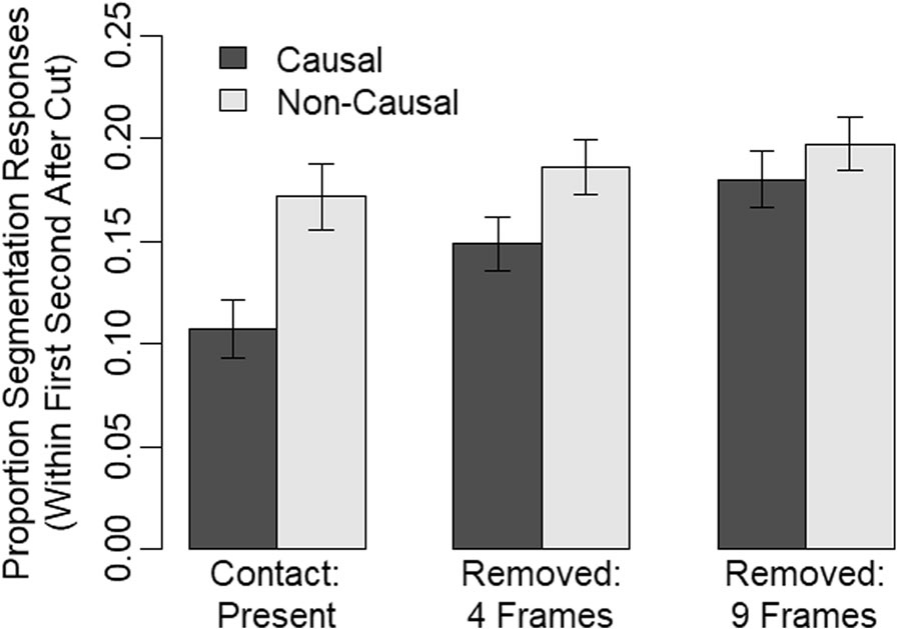
Results of Experiment 4. Error bars indicate the standard error of the mean

As dependent measure, we calculated the proportion of segmentation responses for each participant. That is, we divided the number of clips containing at least one segmentation response in the first second following the cut by the number of all clips for each condition. This provides a measure of how likely participants perceived an event boundary in each condition. We analyzed the proportion of segmentation responses (see Fig. 6) with a two-factorial mixed ANOVA containing the factors causality (causal, non-causal; between) and contact (present, four frames removed, nine frames removed; within). There were significant main effects for causality, *F* (1, 38) = 5.73, *p* = .022, *η*_*p*_^2^ = .13, and contact, *F*(2, 76) = 13.87, *p* < .001, *η*_*p*_^2^ = .27. Thus, segmentation behavior increased with non-causal continuations and the more frames there were removed. Importantly, however, there was also a significant interaction of causality and contact, *F*(2, 76) = 3.17, *p* = .048, *η*_*p*_^2^ = .08, that we further investigated with follow-up *t* tests. There was a significant effect of causality for the contact present trials, *t* (38) = 2.98, *p* = .005, a marginally significant effect for the four frames removed trials, *t* (38) = 2.00, *p* = .053, and a non-significant finding for the nine frames removed trials, *t* (38) = 0.92, *p* = .362. That is, causality influenced segmentation behavior both in the trials where the contact moment was present and in the trials with four frames removed where participants inferred the contact moment as shown by the event-completion effect in our previous experiments presented above. For our control trials with nine frames removed, however, no effect of causality was observed. These results provide the first evidence of a strong link between event completion and event segmentation.

## General discussion

In many situations of daily and professional life it is crucial that people can correctly judge what information they have actually seen, such as with referees or eye-witnesses. However, observers regularly complete missing information during the perception of coherent events. We investigated the process driving event completion in situations of full attention towards the missing information with four experiments. In our first experiment, we found that event completion occurs quickly during event perception. That is, if a causal continuation rather than a non-causal continuation followed missing information, participants false alarmed regardless of whether participants responded immediately during the perception of an event or whether they delayed their responses until after an event. With our second experiment, we investigated whether event completion is caused by predictive perception or backward inferences. Showing a white mask following the missing information resulted in a similar contact-detection performance as non-causal continuations. We thus suggest that event completion is not caused by predictive perception. Instead, backward inferences are drawn once a causal continuation follows missing information. With our third experiment we investigated whether event completion occurs directly during a single trial based on backward inferences or whether participants form expectations based on repeated presentations of the same causality condition. Our results indicate that there was no confounding effect of expectations because event completion occurred equally if causality was manipulated within subjects and trial order was intermixed. This provides further evidence for our conclusion that perceptual gaps are filled in based on backward inferences. Our fourth experiment investigated the link between event completion and event segmentation. We asked participants to perform an event segmentation task while watching our event-completion stimuli. We observed some correspondence between event segmentation and event completion because causal continuations resulted in lower event segmentation responses both when the critical moment was present or when it was absent due to the removal of four frames. That is, participants perceived a more coherent event with causal continuation than non-causal continuation both with the causal link being present and with the causal link being inferred (four-frames-removed condition). This provides converging evidence that participants filled in the perceptual gap when four frames were deleted. Interestingly, the causal continuation did not affect event segmentation if nine instead of four frames were removed. In this case, event segmentation was high in both continuation conditions. This indicates that participants did not infer the causal link if nine frames were removed and, thus, were more likely to perceive two events in both continuation conditions.

Taken together, our results suggest that event completion occurs quickly based on backward inferences. Further, event completion and event segmentation seem to be related, possibly because they both support event model construction during event perception (Magliano et al., 2013). That is, incoming information that is causally linked to the currently active event model is integrated into a coherent event representation. This integration process includes backward inferences filling in the gap between the represented information and incoming information. The conclusion that the completed information is not only perceived but integrated into a mental representation of the current event is supported by the traditional event-completion paradigm that probes memory rather than perception (Brockhoff et al., 2016; Strickland & Keil, 2011). Thus, it seems promising for future research to further investigate the link between event completion and event segmentation. One could, for example, ask the same participants to perform both an event-completion task and an event segmentation task and investigate whether event completion is associated with reduced segmentation on a trial-by-trial basis.

Our findings that event models are constantly updated during event perception have theoretical implications. This is compatible with the structure-building framework (Gernsbacher, 1997) that suggests the presence of a mapping process during the processing of ongoing events. Mapping occurs as long as incoming information activates similar memory nodes as the currently active event model. During mapping, incoming information is integrated with the existing information. Our data suggests that the integration that occurs during mapping includes backward inferences to complete missing information. Doing so, the active event representation is constantly updated through incoming information. This is in contrast to Event Segmentation Theory (Zacks et al., 2007), which proposes that the current event model stored in working memory is not updated by sensory information as long as predictive perceptions match the perceived information. According to EST, global updating is triggered by an error-detection mechanism – in the sense of a complete reset of the present event model – once the event model does not represent real-world observations any more. Thus, our results add to a growing volume of literature suggesting the existence of incremental updating both on a theoretical (Zwaan, Langston, & Graesser, 1995) and an empirical level (Bailey & Zacks, 2015; Huff et al., 2018; Huff, Meitz, & Papenmeier, 2014; Kurby & Zacks, 2012).

Our findings that event completion is caused by backward inferences rather than predictive perception should not be taken as evidence that predictive perception (Zacks et al., 2007) does not exist at all during event perception. In particular, giving participants the task to detect whether the moment of ball contact was present or absent might have increased their uncertainty about the occurrence of ball contacts in our video clips, thus causing a reduction in the generation of perceptual predictions. Our findings rather show that information is quickly integrated into event models during event perception and that this integration of information into coherent event models in working memory is a constructive process based on backward inferences.

Following the event-completion paradigm, we used filmic cuts to manipulate the deletion of information and the presentation of either causal or non-causal continuations. While the use of filmic cuts could be considered to be a limitation of this paradigm, narrative comprehension is hardly influenced by cuts and observers even miss a significant amount of cuts if their main task is the detection of cuts (Smith & Henderson, 2008; Smith & Martin-Portugues Santacreu, 2017). Therefore, we assume that our findings might transfer to natural environments even though we studied event completion with filmic cuts. Nonetheless, we consider it a main challenge of future research to further investigate the processes occurring during the perception of ongoing events (Huff & Papenmeier, 2017). Possible research questions include the completion of information that was missed due to attentional lapses and the completion of information missing due to perceptual constraints such as saccadic suppression (Matin, 1974).

Our findings also provide new practical implications for situations where people make judgments of causal events, such as referees or eye-witnesses. Our results indicate that the causal structure of events biases observers’ responses even when they know what to look for and when they focus their full attention towards this task. This is important because asking people involved in such judgments to pay close attention might not be sufficient to ensure unbiased judgments, particularly if crucial information is not visible by the observer, as it might be the case if information is occluded from the observers’ viewpoint. In our earlier research on event completion (Brockhoff et al., 2016), we argued in favor of an introduction of video replays and a video referee into soccer games because referees might, for example, judge to have seen a foul play that was causally plausible but never present. Our new research shows that event completion occurs quickly based on backward inferences. Watching a replay of a controversial scene might, therefore, not be sufficient to ensure correct decisions, because backward inferences could also quickly occur while watching the replay with full attention. Thus, guidelines for the design and implementation of video reviews should include methods designed to prevent backward inferences from occurring, such as replacing the causal consequence of an action with a white mask. Using slow motion and stopping the controversial action to hide the causal consequence might also support the accurate perception through the prevention of event completion, although the application of technical aids such as slow motion might in itself cause new biases (Spitz, Moors, Wagemans, & Helsen, 2018). The considerations above apply not only to sport games but also to other situations where critical decisions are based on video-taped evidence, such as when using videos in court (Feigenson & Dunn, 2003).

## Abbreviations

EST: Event Segmentation Theory

## Footnotes

1 Although we are confident that the 1-s interval was a good tradeoff for the reasons provided in the main text, one might be concerned that our results relied on this specific time interval. Thus, we ran a re-analysis of our data with the following time intervals: 0.75 s, 1 s, 1.25 s, and 1.5 s. All four analyses provided similar results. In particular, changing the time interval did not change any of the significances reported in the results section, neither for the ANOVA nor for the follow-up *t* tests. The respective analysis scripts are available together with the open data on OSF.

## References

[CR1] Bach P, Schenke KC (2017). Predictive social perception: Towards a unifying framework from action observation to person knowledge. Social and Personality Psychology Compass.

[CR2] Bailey HR, Kurby CA, Giovannetti T, Zacks JM (2013). Action perception predicts action performance. Neuropsychologia.

[CR3] Bailey HR, Zacks JM (2015). Situation model updating in young and older adults: Global versus incremental mechanisms. Psychology and Aging.

[CR4] Brockhoff A, Huff M, Maurer A, Papenmeier F (2016). Seeing the unseen? Illusory causal filling in FIFA referees, players, and novices. Cognitive Research: Principles and Implications.

[CR5] Burke L (1952). On the tunnel effect. Quarterly Journal of Experimental Psychology.

[CR6] Enns JT, Lleras A (2008). What’s next? New evidence for prediction in human vision. Trends in Cognitive Sciences.

[CR7] Feigenson N, Dunn MA (2003). New visual technologies in court: Directions for research. Law and Human Behavior.

[CR8] Freyd JJ, Finke RA (1984). Representational momentum. Journal of Experimental Psychology: Learning, Memory, and Cognition.

[CR9] Gernsbacher MA (1997). Two decades of structure building. Discourse Processes.

[CR10] Graesser AC, Singer M, Trabasso T (1994). Constructing inferences during narrative text comprehension. Psychological Review.

[CR11] Graf M, Reitzner B, Corves C, Casile A, Giese M, Prinz W (2007). Predicting point-light actions in real-time. NeuroImage.

[CR12] Haviland SE, Clark HH (1974). What’s new? Acquiring new information as a process in comprehension. Journal of Verbal Learning and Verbal Behavior.

[CR13] Hayes AE, Freyd JJ (2002). Representational momentum when attention is divided. Visual Cognition.

[CR14] Hubbard TL (2005). Representational momentum and related displacements in spatial memory: A review of the findings. Psychonomic Bulletin & Review.

[CR15] Hudson M, Nicholson T, Ellis R, Bach P (2016). I see what you say: Prior knowledge of other’s goals automatically biases the perception of their actions. Cognition.

[CR16] Huff M, Maurer AE, Brich I, Pagenkopf A, Wickelmaier F, Papenmeier F (2018). Construction and updating of event models in auditory event processing. Journal of Experimental Psychology: Learning, Memory, and Cognition.

[CR17] Huff M, Meitz TGK, Papenmeier F (2014). Changes in situation models modulate processes of event perception in audiovisual narratives. Journal of Experimental Psychology: Learning, Memory, and Cognition.

[CR18] Huff M, Papenmeier F (2017). Event perception: From event boundaries to ongoing events. Journal of Applied Research in Memory and Cognition.

[CR19] Kilner JM, Friston KJ, Frith CD (2007). Predictive coding: An account of the mirror neuron system. Cognitive Processing.

[CR20] Kurby CA, Zacks JM (2012). Starting from scratch and building brick by brick in comprehension. Memory & Cognition.

[CR21] Larsen A, Farrell JE, Bundesen C (1983). Short- and long-range processes in visual apparent movement. Psychological Research.

[CR22] Magliano JP, Kopp K, Higgs K, Rapp DN (2017). Filling in the gaps: Memory implications for inferring missing content in graphic narratives. Discourse Processes.

[CR23] Magliano JP, Larson AM, Higgs K, Loschky LC (2016). The relative roles of visuospatial and linguistic working memory systems in generating inferences during visual narrative comprehension. Memory & Cognition.

[CR24] Magliano JP, Loschky LC, Clinton JA, Larson AM, Miller B, Cutting L, McCardle P (2013). Is reading the same as viewing? An exploration of the similarities and differences between processing text- and visually based narratives. Unraveling the behavioral, neurobiological, and genetic components of reading comprehension.

[CR25] Matin E (1974). Saccadic suppression: A review and an analysis. Psychological Bulletin.

[CR26] Newtson D, Engquist G (1976). The perceptual organization of ongoing behavior. Journal of Experimental Social Psychology.

[CR27] Papenmeier, F. (2018). *powerbydesign: Power Estimates for ANOVA Designs*. R package version 1.0.4. Retrieved from https://CRAN.R-project.org/package=powerbydesign

[CR28] Peirce, J. W. (2007). PsychoPy - Psychophysics software in Python. *Journal of Neuroscience Methods, 162*, 8–13. 10.1016/j.jneumeth.2006.11.017. 10.1016/j.jneumeth.2006.11.017 PMC2018741 17254636

[CR29] Peirce, J. W. (2009). Generating stimuli for neuroscience using PsychoPy. *Frontiers in Neuroinformatics, 2*:10. 10.3389/neuro.11.010.2008. 10.3389/neuro.11.010.2008 PMC2636899 19198666

[CR30] Rauschenberger R, Yantis S (2001). Masking unveils pre-amodal completion representation in visual search. Nature.

[CR31] Richmond LL, Gold DA, Zacks JM (2017). Event perception: Translations and applications. Journal of Applied Research in Memory and Cognition.

[CR32] Schmalhofer F, McDaniel MA, Keefe D (2002). A unified model for predictive and bridging inferences. Discourse Processes.

[CR33] Singer M, Ferreira F (1983). Inferring consequences in story comprehension. Journal of Verbal Learning and Verbal Behavior.

[CR34] Singer M, Halldorson M, Lear JC, Andrusiak P (1992). Validation of causal bridging inferences in discourse understanding. Journal of Memory and Language.

[CR35] Smith TJ, Henderson JM (2008). Edit blindness: The relationship between attention and global change blindness in dynamic scenes. Journal of Eye Movement Research.

[CR36] Smith TJ, Martin-Portugues Santacreu JY (2017). Match-action: The role of motion and audio in creating global change blindness in film. Media Psychology.

[CR37] Spitz J, Moors P, Wagemans J, Helsen WF (2018). The impact of video speed on the decision-making process of sports officials. Cognitive Research: Principles and Implications.

[CR38] Strickland B, Keil F (2011). Event completion: Event based inferences distort memory in a matter of seconds. Cognition.

[CR39] Swallow KM, Zacks JM, Abrams RA (2009). Event boundaries in perception affect memory encoding and updating. Journal of Experimental Psychology: General.

[CR40] Wynn K (1996). Infants’ individuation and enumeration of actions. Psychological Science.

[CR41] Zacks JM, Braver TS, Sheridan MA, Donaldson DI, Snyder AZ, Ollinger JM (2001). Human brain activity time-locked to perceptual event boundaries. Nature Neuroscience.

[CR42] Zacks JM, Speer NK, Reynolds JR (2009). Segmentation in reading and film comprehension. Journal of Experimental Psychology: General.

[CR43] Zacks JM, Speer NK, Swallow KM, Braver TS, Reynolds JR (2007). Event perception: A mind-brain perspective. Psychological Bulletin.

[CR44] Zwaan RA, Langston MC, Graesser AC (1995). The construction of situation models in narrative comprehension: An event-indexing model. Psychological Science.

[CR45] Zwaan RA, Radvansky GA (1998). Situation models in language comprehension and memory. Psychological Bulletin.

